# Soft X-ray tomography analysis of mitochondria dynamics in *Saccharomyces cerevisiae*

**DOI:** 10.1186/s13062-024-00570-2

**Published:** 2024-11-29

**Authors:** Wei-Ling Huang, Chang-Lin Chen, Zi-Jing Lin, Chia-Chun Hsieh, Mo Da-Sang Hua, Chih-Chan Cheng, Tzu-Hao Cheng, Lee-Jene Lai, Chuang-Rung Chang

**Affiliations:** 1 Institute of Biotechnology, National Tsing Hua University, Hsinchu, Taiwan; 2 Experimental Facility Division, National Synchrotron Radiation Research Center, Hsinchu, Taiwan; 3 Institute of Biochemistry and Molecular Biology, National Yang Ming Chiao Tung University, Taipei, Taiwan

**Keywords:** Soft X-ray tomography, Mitochondria, Yeast

## Abstract

**Background:**

Mitochondria are highly dynamic organelles that constantly undergo processes of fission and fusion. The changes in mitochondrial dynamics shape the organellar morphology and influence cellular activity regulation. Soft X-ray tomography (SXT) allows for three-dimensional imaging of cellular structures while they remain in their natural, hydrated state, which omits the need for cell fixation and sectioning. Synchrotron facilities globally primarily use flat grids as sample carriers for SXT analysis, focusing on adherent cells. To investigate mitochondrial morphology and structure in hydrated yeast cells using SXT, it is necessary to establish a method that employs the flat grid system for examining cells in suspension.

**Results:**

We developed a procedure to adhere suspended yeast cells to a flat grid for SXT analysis. Using this protocol, we obtained images of wild-type yeast cells, strains with mitochondrial dynamics defects, and mutant cells possessing distinctive mitochondria. The SXT images align well with the results from fluorescent microscopy. Optimized organellar visualization was achieved by constructing three-dimensional models of entire yeast cells.

**Conclusions:**

In this study, we characterized the mitochondrial network in yeast cells using SXT. The optimized sample preparation procedure was effective for suspended cells like yeast, utilizing a flat grid system to analyze mitochondrial structure through SXT. The findings corresponded with the mitochondrial morphology observed under fluorescence microscopy, both in regular and disrupted dynamic equilibrium. With the acquired image of unique mitochondria in *Δhap2* cells, our results revealed that intricate details of organelles, such as mitochondria and vacuoles in yeast cells, can be characterized using SXT. Therefore, this optimized system supports the expanded application of SXT for studying organellar structure and morphology in suspended cells.

**Supplementary Information:**

The online version contains supplementary material available at 10.1186/s13062-024-00570-2.

## Introduction

Mitochondria, known as the powerhouse of the cell, continuously engage in fission and fusion processes. These activities create a dynamic network that balances to preserve their functionality and integrity [1]. Mitochondrial membrane dynamics are regulated by dynamin-like GTPase, the cytoskeleton, and the endoplasmic reticulum [2–5]. Mitochondria fragment when fission is more frequent or when there is a fusion deficiency, whereas they hyperfuse if fusion increases or a fission defect occurs. Recent studies have revealed several unique mitochondrial structures and derivatives in eukaryotic models under different circumstances, including donut-shaped mitochondria [6], megamitochondria [7, 8], mitochondrial pearling [9], and mitochondrial-derived vesicles (MDVs) [10]. The diverse morphologies of mitochondria have been linked to the equilibrium of dynamic processes influenced by specific signaling pathways that respond to environmental stress [11]. Regulating mitochondrial dynamics is crucial for maintaining cellular energy balance, metabolism, and quality control. Consequently, mitochondrial morphology indicates both dynamic balance and physiological status.

Soft X-ray tomography (SXT) is an advanced imaging technique that enables high-resolution two-dimensional (2D) and three-dimensional (3D) visualization of hydrated cells [12]. Utilizing the specific energy range of soft X-rays (284–543 eV) creates a “water window” due to the differential absorption between carbon and oxygen [13]. This natural contrast allows visualization of cellular structures, such as organelle membranes [14, 15] and distinct biomolecular condensates [16–18], without staining. SXT achieves a spatial resolution of ≤ 50 nm, which is sufficient for examining organellar interactions within cells. Additionally, the sample preparation for SXT involves simply freezing intact cells without chemical fixation or sectioning, which preserves the native state of cells and saves time. The structural characterization of mitochondria using SXT have been performed in various models, including *S. cerevisiae* [19], *C. albicans* [20], and mammalian cells [21–23]. Hence, SXT is a powerful tool for studying mesoscale components, providing detailed insights into mitochondrial structures.

There are two different sample carriers: flat grid system and glass capillary system. Synchrotron facilities around the world predominantly use flat grids as sample carriers for SXT analysis, typically for examining adherent cells [24]. On the other hand, the glass capillary system is utilized for analyzing suspension cells. This system has been employed to explore the cellular structures of various suspension cell models, including *S. pombe*, *S. cerevisiae*, *C. albicans*, and some prokaryotes [25]. However, glass capillary system is currently available at only one synchrotron facility in the United States [26]. Given the high accessibility of SXT facilities with the flat grid system, we aimed to establish a protocol for studying mitochondrial network structure in suspended yeast cells.

The SXT analysis can achieve a spatial resolution of 15–30 nm for 2D imaging and 50 nm for 3D tomography. By optimizing the cell seeding and the freezing processes, we successfully adhered the yeast cells to the grid and analyzed them using SXT. Yeast strains lacking key mitochondrial dynamics factors, such as Dnm1 and Fzo1, were examined to validate the results of this system. Furthermore, the optimized protocol applied to a heme-activated protein (HAP, an upstream transcription factor that activates catabolic derepression) complex-deficient strain resulted in a distinct mitochondrial network morphology, which was different from the typical mitochondrial structure. This finding demonstrates the value of SXT in studying the complexities of yeast mitochondria and sets the stage for using suspension cells on flat grids in future SXT research.

## Materials and methods

### Yeast strains, cultures, and growth conditions

*S. cerevisiae* W303-1 A (*MATa his3Δ leu2Δ trp1Δ ura3Δ*), *Δdnm1* (*MATa his3Δ leu2Δ trp1Δ ura3Δ*,* dnm1::HIS3*), *Δfzo1* (*MATa his3Δ leu2Δ trp1Δ ura3Δ*,* fzo1::HPH*), and *Δhap2* (*MATa his3Δ leu2Δ trp1Δ ura3Δ*,* hap2::KMX6*) strains were used in this study. The *Δdnm1*, *Δfzo1* and *Δhap2* strains were constructed by replacing the targeted genes *DNM1*, *FZO1* and *HAP2* with the PCR-amplified *HIS3*, *HPH* and *KMX6* cassettes in the parental strain, respectively (Supplementary Table 1) [27]. Genotyping of the deletion strains was confirmed using PCR. Yeast cells were grown in YP medium (1% (w/v) yeast extract (2005, Cyrusbioscience) and 2% (w/v) peptone (90000-264, BD)) with 2% (w/v) dextrose (101-14431-43-7, Cyrusbioscience) at 30 °C. Yeast cells were grown to the log phase (optical density of 0.6 to 0.8 at 600 nm) and stationary phase (48 h after the log phase) for use in this study.

### Premarking of gold grid preparation

Quantifoil^®^ R2/2 Au G200F1 finder grids (#N1-C16nAuG1-01, Micro Tools GmbH, Germany) were utilized for cell seeding. To increase the cell adhesion efficiency, the gold grids were glow discharged (15 mA, 25 s) via a PELCO easiGLOW™ system (Ted Pella, Inc., CA) to increase the surface hydrophilicity. To premark the gold grids, 20 µl of 100 nm nano-gold colloid solution (EM. GC100, BBI Solutions, Crumlin, UK) was dropped onto a parafilm, and the discharged gold grid, with the carbon side facing upward, was submerged in the nano-gold solution for 1 h. After incubation, the premarked gold grids were subsequently transferred onto filter paper to remove excess water, and an additional 10 µl of the nano-gold solution was immediately added to the grids to increase the number of gold nanoparticles on the grids. Afterward, the R2/2 gold grids were dried overnight in a dryer set at 5% relative humidity (RH) for further experiments.

### Sample preparation

Log-phase and stationary-phase yeast cells were adjusted to an optical density of 1.0 at the wavelength of 600 nm with the culture medium. Yeast cells were stained with 70 nM µM DiOC_6_(3) (3,3’-dihexyloxacarbocyanine iodide, #D273, Thermo Fisher) for 15 min. Next, they were washed and resuspended in PBS, pH of 7. The nano-gold solution was washed and resuspended in PBS. The nano-gold solution was subsequently mixed with the yeast suspension at a 1:1 ratio. For sample grid preparation, 1 and 5 µl of the mixture were loaded onto the gold and carbon sides of the premarked gold grid, respectively. To prepare cryo-sample, the premarked sample grid was subsequently blotted from the gold side with the filter paper to remove excess water and plunged into liquid ethane by a plunge freezer EM GP2 (Leica, Germany). Prior to SXT imaging, we applied a Cryo-Correlative Microscopy Stage CMS196V3 (Linkam, UK) combined with a Zeiss Axio Imager A2 fluorescence microscope (Carl Zeiss, Germany) to screen for regions of interest of the sample grids. The cryo-samples were preserved in liquid nitrogen until SXT imaging.

### Soft X-ray tomography

SXT was performed at the Taiwan Photon Source 24 A (TPS 24A) at the National Synchrotron Radiation Research Center (NSRRC, Hsinchu, Taiwan). The cryo-samples were transferred into a load‒lock vacuum system and transported to the end station of the TPS24A. For SXT, tilt images were collected with a photon energy of 520 eV using full-field transmission soft X-ray microscopy. Projection images were continuously acquired in 1° increments around the axis of rotation (from − 68° to + 68°), with a maximum of 137 images per dataset. The tomographic images were adjusted based on the background of the flat-field images using MATLAB. All tomographic datasets were aligned by fiducial markers of 100 nm nano-gold particles and reconstructed using IMOD [28]. Mitochondria visualized by cryo-stage fluorescence microscopy were correlated with the reconstructed images and adjusted through Fiji (ImageJ) [29].

### Fluorescence microscopy

Yeast cells were grown to either log phase or stationary phase for imaging purposes. Mitochondria were detected by staining with DiOC_6_(3) or by transforming with the plasmid pVT100U-mtGFP. Imaging was performed using a 63× objective (Zeiss Plan-ApoChromat DIC M27) on Zeiss Axioskop 2 mot plus fluorescence microscope (Carl Zeiss, Oberkochen, Germany) with an integrated camera system controlled by Zeiss Zen software. Image analysis was conducted using Zen software.

## Results

### Development of an optimized flat grid sample preparation procedure of yeast cells for soft X-ray tomography

**Fig. 1:**
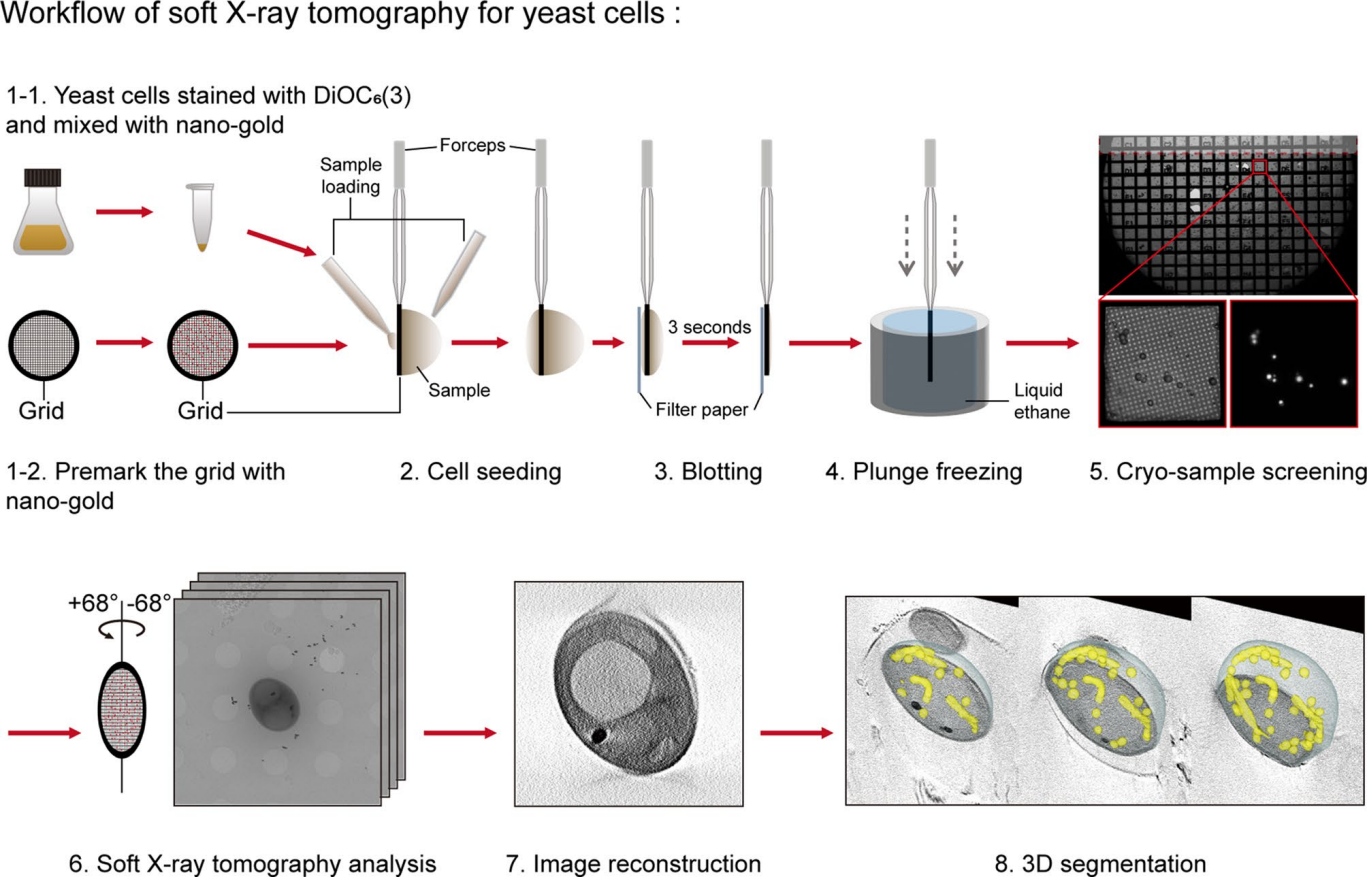
Workflow of soft X-ray tomography for yeast cells. Yeast cells were stained with mitochondria dye DiOC_6_(3) and mixed with 100 nm nano-gold particles. Glow discharged grids were premarked with nano-gold particles. Yeast and nano-gold samples were seeded on both sides of the grids. Next, grids were blotted with filter paper and frozen in liquid ethane using a plunge freezer. After freezing, cryo-samples were screened using a cryo-stage fluorescence microscope to select the regions of interest for further SXT analysis. IMOD and Fiji (ImageJ) software were used to reconstruct SXT images. IMOD software was used to build 3D segmented models

Preparing yeast cells for SXT using the flat grid system presents several hurdles, including the adhesion of yeast cells on the grid, management of ice thickness during the freezing process, control of yeast cell density, and consistent distribution of fiducial markers on the grid. To overcome these hurdles, we developed a modified sample preparation protocol for yeast cells, as demonstrated in Fig. 1. Here, we delineated the optimized conditions for cryo-sample preparation.

We utilized gold grids with carbon films (Quantifoil^®^ R2/2 Au G200F1 finder grids) as the platform for cell seeding. To attach the sample to the grid, previous studies applied poly-L-lysine (PLL) to enhance the attachment of adherent cells [30, 31]. PLL is also applied to enhance yeast cell attachment on the surface of glass slides for microscopy [32]. In this study, we adhered the yeast cells on the grid without additional coating of the PLL to the grid surface. Yeast cells were mixed with the fiducial markers of 100 nm nano-gold particles. A mixture containing yeast cells and nano-gold particles was applied on both sides of the premarked grid that underwent a glow discharge process. Filter paper blotting was executed from the gold side of the grid to remove excess liquid. This action generated a capillary force that drew the liquid from the carbon side, thereby depositing the yeast cells onto the gold grid (Fig. 1). Loading samples and blotting on the gold side ensured the efficacy of the subsequent blotting process, which allowed the suspended yeast cells to adhere to the grid.

The subsequent freezing process creates an ice layer that covers the sample. The thickness of the ice layer determines the sample protection from soft X-rays and predominates the contrast of the cell images. The sample is damaged by soft X-rays when the ice layer is too thin, whereas an excessively thick ice layer can lead to blurring of the cell image. Therefore, the management of ice thickness during the freezing process is critical. We found that a 3-second blotting time was optimal for achieving the desired ice thickness of yeast cryo-samples (Fig. 1). This blotting time was subsequently adopted in all our sample preparations.

During the SXT analysis, the sample grids were rotated to capture the cell images at different angles (from − 68° to + 68°) at an interval of 1 degree. All tomographic datasets were making a stack image and further for reconstruction and segmentation (Fig. 1). When yeast cells are densely packed, short intercell distances increase the probability of cells obscuring one another during rotation in high tilt angle, which increases the thickness of the cells and reduces the depth of the soft X-ray penetration, resulting in compromised image quality. Accordingly, controlling the density of yeast cells is important. It can minimize cell stacking and allow the sample images to be acquired to high tilt angles to reduce the missing wedge during the image reconstruction. We found that an optical density of 600 nm, approximately 1.0, was optimal for SXT analysis.

**Fig. 2:**
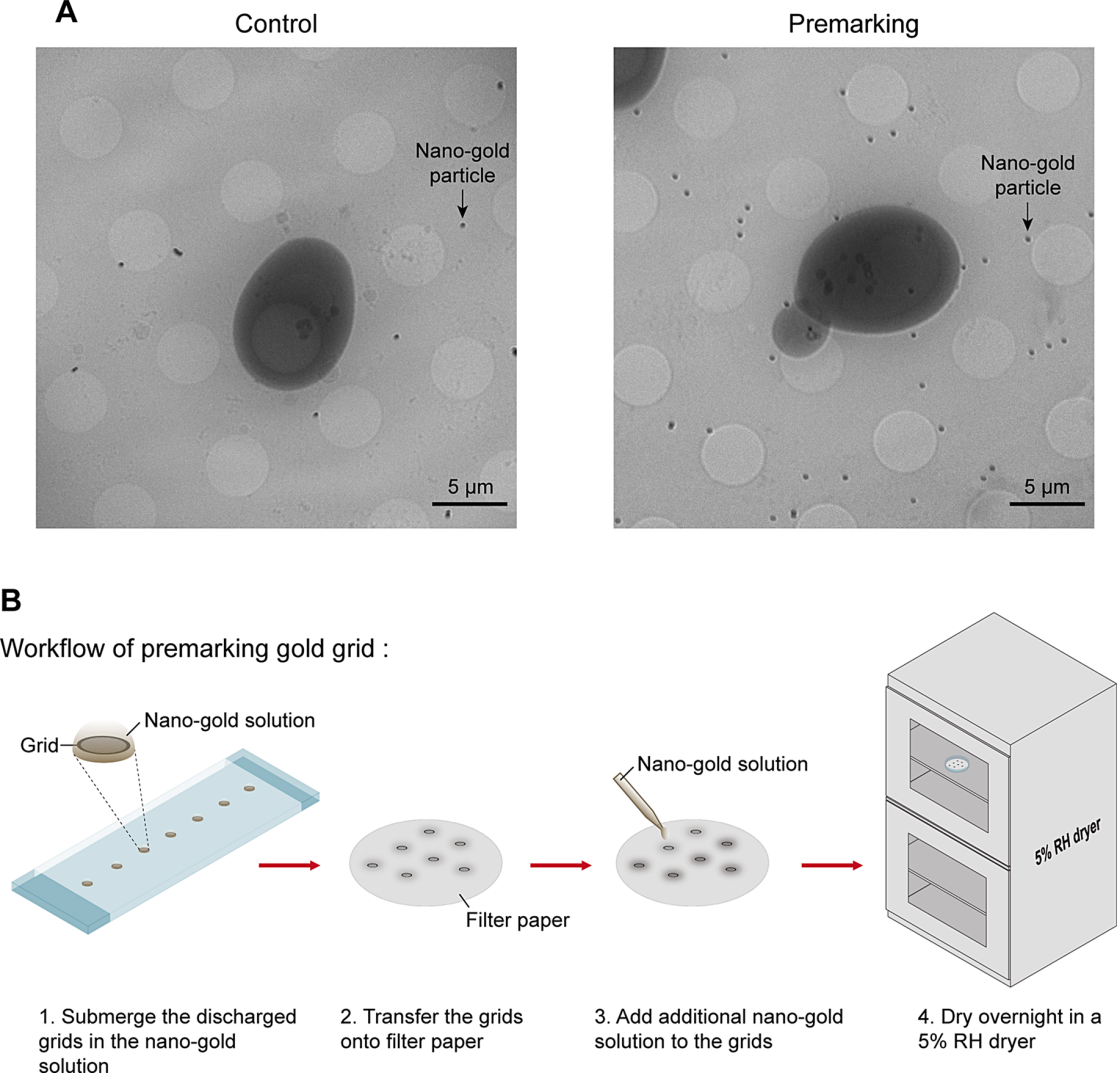
Premarking process improves the distribution of nano-gold particles on the grid. (**A**) Representative SXT images of R2/2 200-mesh grid without (control, left) and with (premarking, right) pre-incubation of 100 nm nano-gold particles. Arrow indicates nano-gold particle. Scale bar: 5 μm. (**B**) Illustrated workflow of premarking process. Glow discharged grids were submerged in the nano-gold solution dropped onto a parafilm-covered glass slide. After 1-hour incubation, grids were transferred onto filter paper. An additional nano-gold solution was added to the grids. Next, the premarked gold grids were dried overnight in a 5% relative humidity (RH) dryer for further experiments

The 100 nm nano-gold particles were used as fiducial markers to facilitate SXT image alignment and reconstruction. The addition of nano-gold particles to yeast samples immediately before plunge freezing caused no uniform dispersion on the grid (Fig. 2A). To overcome this issue, we pre-incubated the grids in the nano-gold particle solution and subsequently dried them in a 5% relative humidity (RH) dryer. This step was named “premarking” (Fig. 2B). The results showed that only a few fiducial markers were around the yeast cell in control group without the premarking process; however, many fiducial markers were homogeneously spread around the yeast cell with the premarking process. The premarking step ensured an increased amount of gold particles and a more uniform distribution of nano-gold particles on the grid (Fig. 2A). This approach enhances the image reconstruction quality of the SXT.

### Examining wild-type yeast mitochondrial morphology using soft X-ray tomography with flat grid sample preparation procedure

**Fig. 3:**
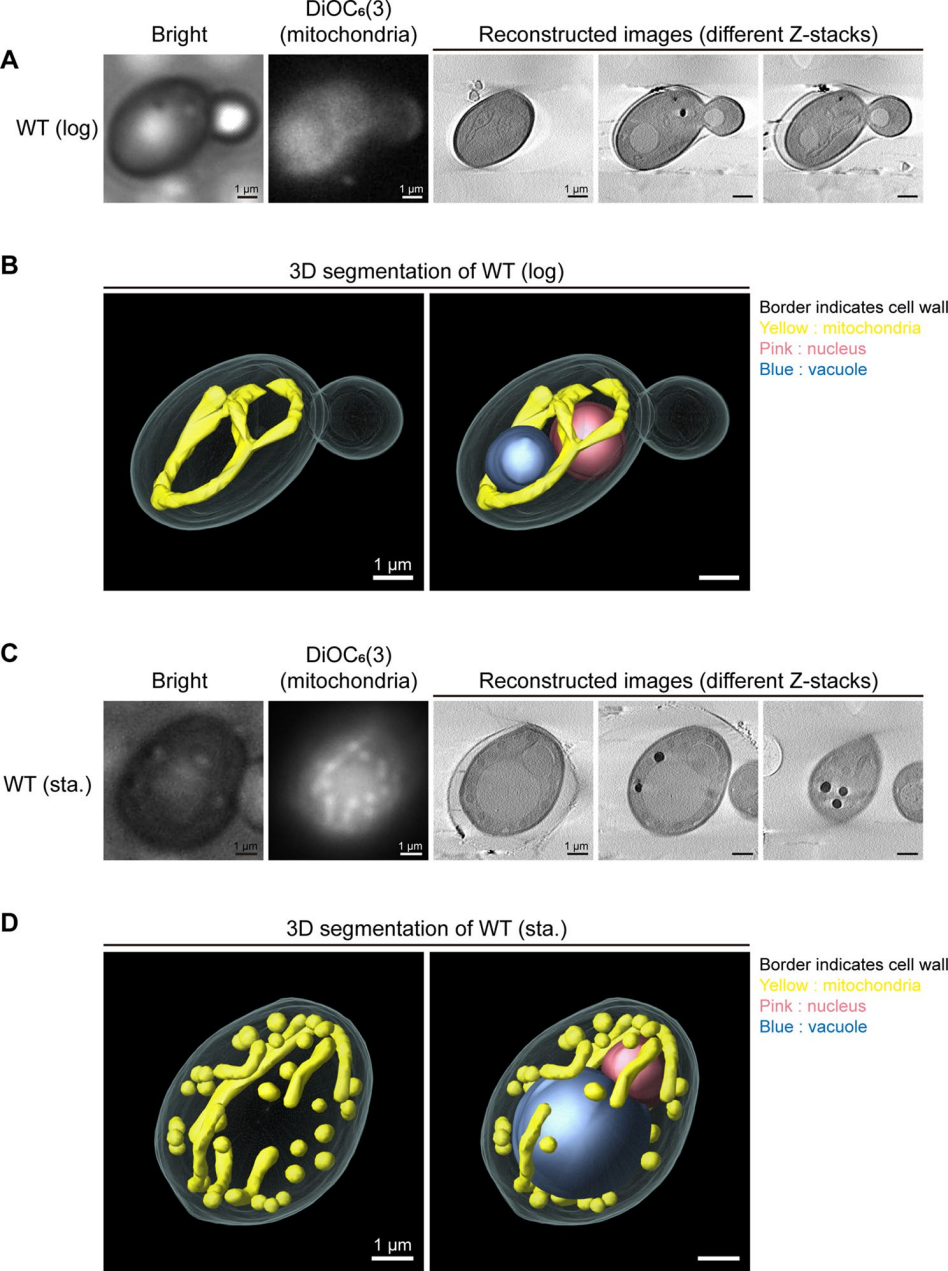
Soft X-ray tomographic analysis of mitochondrial structure and intracellular distribution in yeast cells. (**A**) Mitochondria of wild-type (WT) yeast in log phase (log) were stained with DiOC_6_(3) and examined by cryo-stage fluorescence microscope for recognition of mitochondrial localization before SXT. The localizations of mitochondria visualized by cryo-stage fluorescence microscope were correlated with the reconstructed images of SXT results. (**B**) 3D segmented models of log-phase WT yeast cell was built from the reconstructed images in panel A. The mitochondria in the daughter cell were not shown because of focusing issues during image reconstruction. (**C**) Mitochondria in stationary-phase (sta.) WT yeast was stained with DiOC_6_(3) and examined by cryo-stage fluorescence microscope. Mitochondria visualized by cryo-stage fluorescence microscope were correlated with the reconstructed images of SXT. (**D**) 3D segmented models of stationary-phase WT yeast was built from the reconstructed images in panel C. Scale bar: 1 μm

We examined the organelle structure in wild-type yeast cells using the sample preparation procedure mentioned above. Mitochondria of wild-type yeast cells in the log phase growth have been proved to form tubular networks using fluorescence microscopy (Supplementary figure S1). Additionally, our previous studies revealed that mitochondrial morphology changes from tubular form to fragmented form as yeast cells transition from log phase to stationary phase [4, 33]. To evaluate the feasibility of SXT for studying yeast mitochondrial network morphology, we examined mitochondria of log-phase and stationary-phase wild-type yeast cells. Yeast cells were labeled with DiOC_6_(3), a mitochondrial membrane potential-dependent dye, for the cryo-sample screening process. The cryo-samples were screened using a Cryo-Correlative Microscopy Stage to select the regions of interest for SXT analysis. SXT was performed at the Taiwan Photon Source 24 A beamline of the National Synchrotron Radiation Research Center. After the image reconstruction of SXT, the membrane boundaries of organelles in yeast cells were clearly visible for annotation. The mitochondrial network structure of log-phase yeast cells appeared tubular, which was similar to the fluorescence microscopy images (Fig. 3A). Additionally, a 3D segmented model view of log-phase yeast cell provided a smooth, linear mitochondrion with few branches near the plasma membrane (Fig. 3B; an additional movie file shows this in more detail [see Additional file 1]). To further verify our procedure, we examined mitochondrial structures of stationary yeast cells under post-catabolic repression condition. Stationary cells possessed fragmented mitochondria under fluorescence microscopy [33], and the reconstructed SXT images and 3D segmented model also demonstrated that stationary yeast cells contained short and rounded mitochondria in contrast to log-phase cells (Fig. 3C and D; an additional movie file shows this in more detail [see Additional file 2]). The 3D segmented model clearly depicted the spatial organization of the vacuole, nucleus, and individual mitochondria within the cell (Fig. 3). These results validated the flat grid system of SXT for analyzing mitochondrial morphology in suspension yeast cells.

### Visualizing unique mitochondrial network morphology via soft X-ray tomography

In yeast, Dnm1 and Fzo1 are the primary factors regulating dynamic fission and fusion events of mitochondria [34]. Deletion of *DNM1* results in defective mitochondrial fission and hyperfused mitochondria [35]. In contrast, deletion of *FZO1* impairs mitochondrial fusion, leading to fragmented mitochondria [36]. Deletion of either *DNM1* or *FZO1* has been demonstrated to diminish mitochondrial respiration and increase mitochondrial DNA structural variants [37]. Hyperfused mitochondria depicts the dynamics balance shifting towards fusion, and vice versa.

**Fig. 4:**
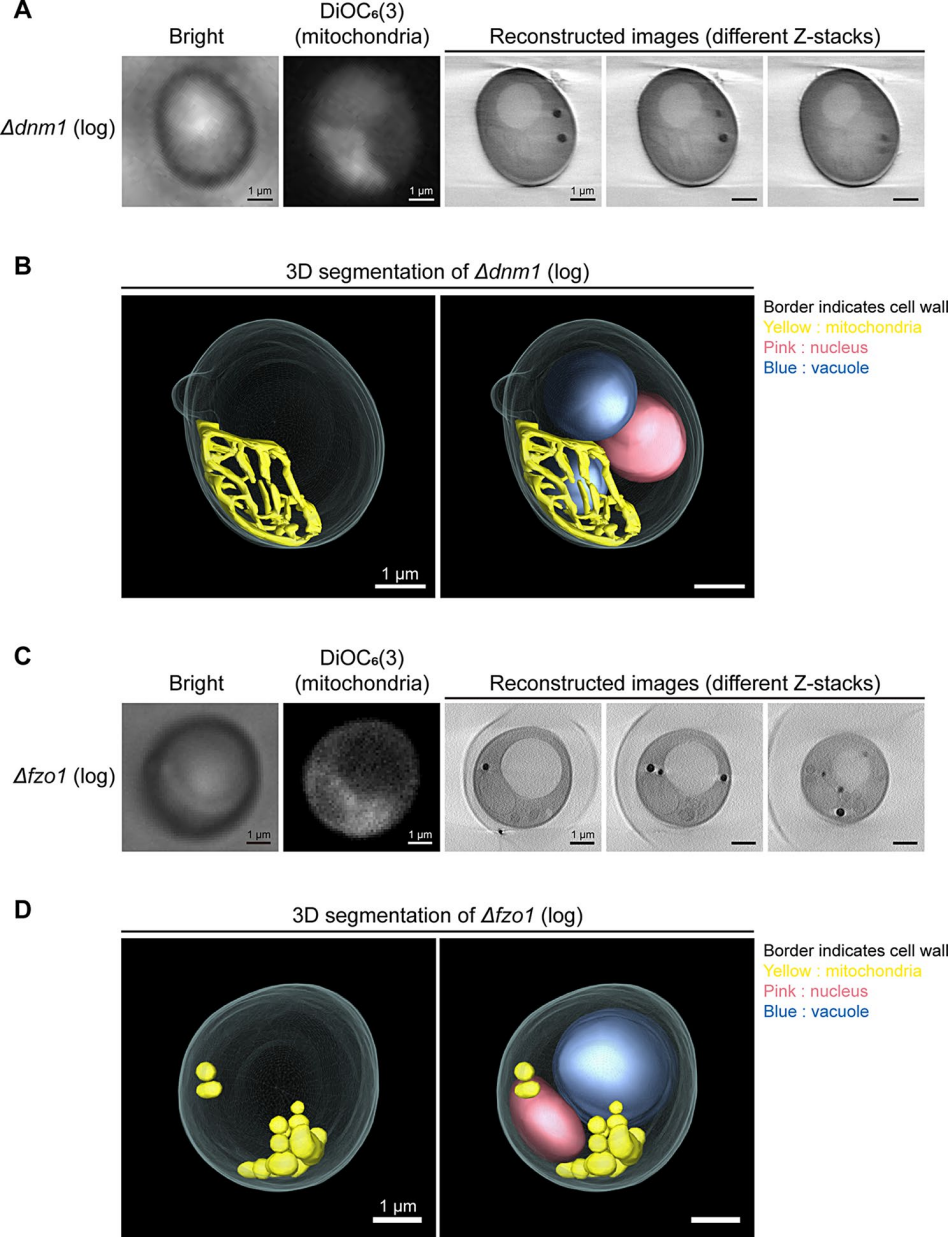
Soft X-ray tomographic analysis of mitochondrial structure in fission/fusion defect yeast cells. (**A**) Mitochondria of *Δdnm1* yeast in log phase (log) were stained with DiOC_6_(3) to detect mitochondrial localization before SXT. Hyperfused mitochondria visualized by cryo-stage fluorescence microscope were correlated with the reconstructed images by SXT. (**B**) 3D segmented models of *Δdnm1* yeast was built from the reconstructed images in panel A. (**C**) Mitochondria of log-phase (log) *Δfzo1* yeast were stained with DiOC_6_(3) to recognize mitochondrial localization before SXT. Fragmented mitochondria visualized by cryo-stage fluorescence microscope were correlated with the reconstructed images by SXT. (**D**) 3D segmented models of *Δfzo1* yeast was built from the reconstructed images in panel C. Scale bar: 1 μm

To determine whether hyperfused and fragmented mitochondrial networks can be visualized by SXT, we prepared samples of *Δdnm1* and *Δfzo1* for SXT analysis. Staining with DiOC_6_(3) confirmed the expected mitochondrial morphology phenotypes in the *Δdnm1* and *Δfzo1* strains using fluorescence microscopy (Fig. 4A and C). The SXT-reconstructed images revealed that *Δdnm1* cells exhibited an elongated and interconnected mitochondrial network (Fig. 4A). Additionally, the 3D segmented model provided a more comprehensive view of this hyperfused structure (Fig. 4B; an additional movie file shows this in more detail [see Additional file 3]). Contrarily, the SXT results of *Δfzo1* strain displayed fragmented mitochondrial morphology (Fig. 4C). The 3D segmented model of *Δfzo1* cells further demonstrated fragmented mitochondria with aggregation in certain areas of the cytoplasm (Fig. 4D; an additional movie file shows this in more detail [see Additional file 4]).

### Applying soft X-ray tomography to examine mitochondrial network in *Δhap2* cells

**Fig. 5:**
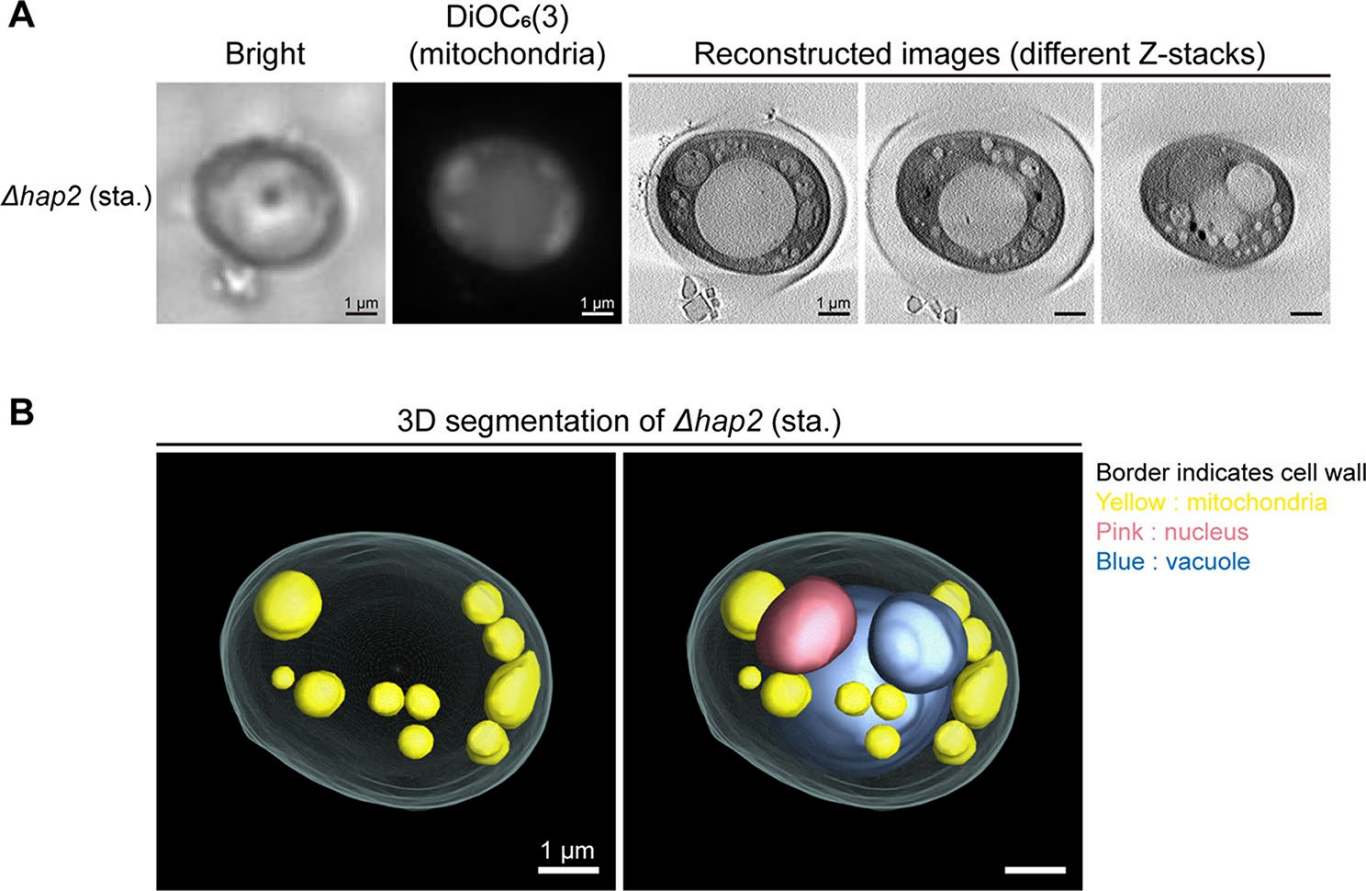
Soft X-ray tomographic analysis of mitochondrial structure in *Δhap2* yeast cells. (**A**) Stationary-phase (sta.) *Δhap2* yeast was stained with DiOC_6_(3) for visualizing mitochondria. Mitochondrial localization, as examined by cryo-stage fluorescence microscope, was correlated with the reconstructed images of SXT. (**B**) Reconstructed images of *Δhap2* were used to build 3D segmented models. Scale bar: 1 μm

Previous reports have indicated that the HAP complex is an upstream transcription factor that activates catabolic derepression in *S. cerevisiae*, playing a crucial role in mitochondrial respiration and biogenesis [38, 39]. However, changes in the mitochondrial dynamics of HAP defects during catabolic derepression have not been reported. We used fluorescence microscopy to classify the mitochondrial morphology of *Δhap2* cells in stationary phase. The results demonstrated that mitochondria in *Δhap2* cells became globular, in contrast to the fragmented mitochondria found in wild-type cells (Figs. 3C and 5A, and Supplementary figure S1). The statistic results indicated that majority of *Δhap2* cells contain globular mitochondria (Supplementary figure S2). We then examined *Δhap2* cells with the procedure as wild-type and dynamic defect cells to verify the application of SXT in characterizing the morphology of mitochondria. The SXT-reconstructed images revealed that *Δhap2* stationary cells possessed globular mitochondria without affecting the vacuole or nucleus (Fig. 5A). The 3D segmented model also demonstrated globular mitochondrial structures in *Δhap2* cells (Fig. 5B; an additional movie file shows this in more detail [see Additional file 5]). Although the detailed molecular mechanism underlying these changes in mitochondrial morphology requires further clarification, these results indicate that SXT is an advanced method for studying mitochondrial dynamics. SXT can offer detailed insights into the structural differences that are not discernible using fluorescence microscopy.

## Discussion

Most synchrotron facilities worldwide primarily utilize the flat grid system for SXT analysis [24, 40–42]. In this study, we delineated the optimized SXT protocol for obtaining yeast samples with flat grid system. Our method provides practical information for the users of these facilities, especially when working with suspension cells such as bacteria (*E. coli*), yeasts (*S. cerevisiae*, *S. pombe*, and *C. albicans*), and even lymphocytes. The other system for suspension cells involves using glass capillaries to hold the cells. The capillary system has advantages for full-angle rotation during SXT and the linear absorption coefficient (LAC) for organelle identification. However, this glass capillary technique for SXT analysis is available only at the Advanced Light Source, Berkeley, United States [26]. This flat grid sample preparation method we provided requires no additional capillary system for suspension cells.

Apart from the optimized conditions in our protocol, several concerns remain regarding sample preparation on the grid. First, regarding the grid pretreatment before cell seeding, we applied no poly-L-lysine (PLL) coating. Instead, we used a glow discharge procedure to increase the surface hydrophilicity before the yeast cells were seeded. This procedure resulted in the uniform distribution of yeast cells on the grid. Additionally, the grid without PLL eliminated nonspecific binding during sample preparation. The effect of an additional coating of PLL on the attachment of other suspension cell types requires further investigation. Second, we assessed various blotting times to determine the optimal ice thickness for cryo-samples. The thickness of the ice layer predominates the contrast of cell images. Our findings indicate that a 3-second blotting time before plunge freezing creates the desired ice thickness for the “water window” of SXT [13]. Blotting for more than 3 s may remove too much solution, drying the grid excessively and preventing the formation of an advisable ice layer during freezing. Conversely, a shorter blotting time would leave excessive solution, resulting in an uneven ice layer on the grid and reducing SXT quality. Third, compared with that of mammalian cells, the cell wall of yeast cells increases the carbon content of cell barrier. This property potentially reduces soft X-ray penetration, leading to lower contrast in SXT images. Even though our image quality is sufficient to visualize most membrane-bound organelles, removing cell wall through mild lyticase treatment in sample preparation can further improve image clarity.

Fluorescence microscopy is widely used for elucidating organelle structure due to its rapid sample generation and simple imaging process. Characterizing mitochondrial morphology can be easily conducted by staining with fluorescent markers, such as DiOC_6_(3) or MitoTracker. Nonetheless, the resolution limit of fluorescence microscopy prevents detailed visualization of mitochondrial structures and their spatial positions. To pursuit higher resolution, transmission electron microscopy (TEM) provides detailed information about the complex internal membrane structures of mitochondria [43]. However, TEM requires chemical fixation and sectioning for sample preparation, which can introduce artifacts and distort cellular structures. SXT offers significant advantages over fluorescence microscopy and complements TEM. SXT provides better imaging of intact cells, avoiding the artifacts associated with the preparation process in TEM. While TEM achieves the highest resolution, SXT surpasses fluorescence microscopy in avoiding cytotoxicity from chemical staining or fluorescence proteins, allowing for more accurate visualization of mitochondrial morphology and internal structures in their native state [44]. Additionally, SXT can provide spatial positioning of different organelles in three-dimensional images. Therefore, SXT serves as a powerful method for studying organelle structures without the drawbacks of delicate handling encountered when doing TEM and fluorescence microscopy.

To strengthen the utility of SXT, integrating deep learning techniques for SXT segmented datasets and identifying organelle shapes is a promising approach. Accurately detecting organellar shapes, such as mitochondrial network morphology, can reflect the dynamic status among organelles. By training computational networks on segmented SXT images, more accurate and automated identification of various organelles can be achieved. Recent publications have demonstrated this approach with mammalian cell samples, revealing the possibility of analyzing SXT data with automated pipelines [45, 46]. In addition, the development of cell-permeable compounds for organelle labeling can further aid in organelle identification using SXT. These compounds selectively bind to specific organelles and are detectable in SXT images [47]. By leveraging deep learning and chemical labeling techniques, the capabilities of SXT for future biological research can be significantly enhanced.

## Conclusions

In this study, we optimized the SXT protocol for yeast samples with flat grid system. We demonstrated that the mitochondria morphology of wild-type yeast cells as tubular form in log phase and fragmented form in stationary phase. Specific hyperfused and fragmented mitochondria in *DNM1* and *FZO1* deletions, respectively, were further demonstrated. Additionally, unique globular mitochondrial structure in stationary *Δhap2* cells was identified using our method. These results unveil the value of our method for studying mitochondria in yeast. Therefore, our findings indicate the potential of flat grid system for studying suspension cells.

## Electronic supplementary material

Below is the link to the electronic supplementary material.

Supplementary Material 1: Additional file 1: Three-dimensional segmented model of log-phase wild-type yeast cell. Border indicates cell wall. Mitochondria: yellow; nucleus: pink; vacuole: blue. Scale bar: 1 μm.

Supplementary Material 2: Additional file 2: Three-dimensional segmented model of stationary-phase wild-type yeast cell. Border indicates cell wall. Mitochondria: yellow; nucleus: pink; vacuole: blue. Scale bar: 1 μm.

Supplementary Material 3: Additional file 3: Three-dimensional segmented model of log-phase *Δdnm1* yeast cell. Border indicates cell wall. Mitochondria: yellow; nucleus: pink; vacuole: blue. Scale bar: 1 μm.

Supplementary Material 4: Additional file 4: Three-dimensional segmented model of log-phase *Δfzo1* yeast cell. Border indicates cell wall. Mitochondria: yellow; nucleus: pink; vacuole: blue. Scale bar: 1 μm.

Supplementary Material 5: Additional file 5: Three-dimensional segmented model of stationary-phase *Δhap2* yeast cell. Border indicates cell wall. Mitochondria: yellow; nucleus: pink; vacuoles: blue. Scale bar: 1 μm.

Supplementary Material 6: Additional file 6: Supplementary table S1, Supplementary figure S1 and Supplementary figure S2

## Data Availability

No datasets were generated or analysed during the current study.
