# Supplementary material for: Soft X-ray tomography analysis of mitochondria dynamics in Saccharomyces cerevisiae
Source: Biol Direct. 2024 Nov 29;19:126. doi: 10.1186/s13062-024-00570-2 (PMC11607810; doi:10.1186/s13062-024-00570-2)
Supplement: Supplementary file 6 — Supplementary Material 6: Additional file 6: Supplementary table S1, Supplementary figure S1 and Supplementary figure S2 [file 13062_2024_570_MOESM6_ESM.pdf]

Supplementary Table

Table S1. Cassette sequence used in this study.

Cassette	Sequence (5’-NNN-3’)
KANMX6	GACATGGAGGCCCAGAATACCCTCCTTGACAGTCTTGACG<br>TGCGCAGCTCAGGGGCATGATGTGACTGTCGCCCGTACAT<br>TTAGCCCATACATCCCCATGTATAATCATTTGCATCCATACA<br>TTTTGATGGCCGCACGGCGCGAAGCAAAAATTACGGCTCC<br>TCGCTGCAGACCTGCGAGCAGGGAAACGCTCCCCTCACA<br>GACGCGTTGAATTGTCCCCACGCCGCGCCCCTGTAGAGA<br>AATATAAAAGGTTAGGATTTGCCACTGAGGTTCTTCTTTCA<br>TATACTTCCTTTTAAAATCTTGCTAGGATACAGTTCTCACA<br>TCACATCCGAACATAAACAACCATGGGTAAGGAAAAGACT<br>CACGTTTCGAGGCCGCGATTAAATTCCAACATGGATGCTG<br>ATTTATATGGGTATAAATGGGCTCGCGATAATGTCGGGCAA<br>TCAGGTGCGACAATCTATCGATTGTATGGGAAGCCCGATG<br>CGCCAGAGTTGTTTCTGAAACATGGCAAAGGTAGCGTTG<br>CCAATGATGTTACAGATGAGATGGTCAGACTAAACTGGCT<br>GACGGAATTTATGCCTCTTCCGACCATCAAGCATTTTATCC<br>GTACTCCTGATGATGCATGGTTACTCACCACTGCGATCCC<br>CGGCAAAACAGCATTCCAGGTATTAGAAGAATATCCTGAT<br>TCAGGTGAAAATATTGTTGATGCGCTGGCAGTGTTCCTGC<br>GCCGGTTGCATTCGATTCCTGTTTGTAATTGTCCTTTTAAC<br>AGCGATCGCGTATTTCGTCTCGCTCAGGCGCAATCACGAA<br>TGAATAACGGTTTGGTTGATGCGAGTGATTTTGATGACGA<br>GCGTAATGGCTGGCCTGTTGAACAAGTCTGGAAAGAAAT<br>GCATAAGCTTTTGCCATTCTCACCGGATTCAGTCGTCACT<br>CATGGTGATTTCTCACTTGATAACCTTATTTTTGACGAGGG<br>GAAATTAATAGGTTGTATTGATGTTGGACGAGTCGGAATC<br>GCAGACCGATACCAGGATCTTGCCATCCTATGGAACTGCC<br>TCGGTGAGTTTTCTCCTTCATTACAGAAACGGCTTTTTCA<br>AAAATATGGTATTGATAATCCTGATATGAATAAATTGCAGT<br>TTCATTTGATGCTCGATGAGTTTTTCTAATCAGTACTGACA

	ATAAAAAGATTCTTGTTTTCAAGAACTTGTCATTTGTATAG<br>TTTTTTTATATTGTAGTTGTTCTATTTTAATCAAATGTTAGC<br>GTGATTTATATTTTTTTTCGCCTCGACATCATCTGCCCAGA<br>TGCGAAGTTAAGTGCGCAGAAAGTAATATCATGCGTCAAT<br>CGTATGTGAATGCTGGTCGCTATACTG
**HPHMX6**	GACATGGAGGCCCAGAATACCCTCCTTGACAGTCTTGACG<br>TGCGCAGCTCAGGGGCATGATGTGACTGTCGCCCGTACAT<br>TTAGCCCATACATCCCCATGTATAATCATTTGCATCCATACA<br>TTTTGATGGCCGCACGGCGCGAAGCAAAAATTACGGCTCC<br>TCGCTGCAGACCTGCGAGCAGGGAAACGCTCCCCTCACA<br>GACGCGTTGAATTGTCCCCACGCCGCGCCCCTGTAGAGA<br>AATATAAAAGGTTAGGATTTGCCACTGAGGTTCTTCTTTCA<br>TATACTTCCTTTTAAAATCTTGCTAGGATACAGTTCTCACA<br>TCACATCCGAACATAAACAACCATGGGTAAAAAGCCTGAA<br>CTCACCGCGACGTCTGTCGAGAAGTTTCTGATCGAAAAGT<br>TCGACAGCGTCTCCGACCTGATGCAGCTCTCGGAGGGCG<br>AAGAATCTCGTGCTTTCAGCTTCGATGTAGGAGGGCGTGG<br>ATATGTCCTGCGGGTAAATAGCTGCGCCGATGGTTTCTAC<br>AAAGATCGTTATGTTTATCGGCACTTTGCATCGGCCGCGC<br>TCCCGATTCCGGAAGTGCTTGACATTGGGGAATTCAGCGA<br>GAGCCTGACCTATTGCATCTCCCGCCGTGCACAGGGTGTC<br>ACGTTGCAAGACCTGCCTGAAACCGAACTGCCCGCTGTT<br>CTGCAGCCGGTCGCGGAGGCCATGGATGCGATCGCTGCG<br>GCCGATCTTAGCCAGACGAGCGGGTTCGGCCCATTCGGA<br>CCGCAAGGAATCGGTCAATACACTACATGGCGTGATTTCA<br>TATGCGCGATTGCTGATCCCCATGTGTATCACTGGCAAAC<br>TGTGATGGACGACACCGTCAGTGCGTCCGTCGCGCAGGC<br>TCTCGATGAGCTGATGCTTTGGGCCGAGGACTGCCCCGA<br>AGTCCGGCACCTCGTGCACGCGGATTTCGGCTCCAACAA<br>TGTCCTGACGGACAATGGCCGCATAACAGCGGTCATTGAC<br>TGGAGCGAGGCGATGTTCGGGGATTCCCAATACGAGGTC<br>GCCAACATCTTCTTCTGGAGGCCGTGGTTGGCTTGTATGG<br>AGCAGCAGACGCGCTACTTCGAGCGGAGGCATCCGGAGC

	**TTGCAGGATCGCCGCGGCTCCGGGCGTATATGCTCCGCAT<br>TGGTCTTGACCAACTCTATCAGAGCTTGGTTGACGGCAAT<br>TTCGATGATGCAGCTTGGGCGCAGGGTCGATGCGACGCA<br>ATCGTCCGATCCGGAGCCGGGACTGTCGGGCGTACACAA<br>ATCGCCCGCAGAAGCGCGGCCGTCTGGACCGATGGCTGT<br>GTAGAAGTACTCGCCGATAGTGGAAACCGACGCCCCAGC<br>ACTCGTCCGAGGGCAAAGGAATAATCAGTACTGACAATAA<br>AAAGATTCTTGTTTTCAAGAACTTGTCATTTGTATAGTTTT<br>TTTATATTGTAGTTGTTCTATTTTAATCAAATGTTAGCGTGA<br>TTTATATTTTTTTTCGCCTCGACATCATCTGCCCAGATGCG<br>AAGTTAAGTGCGCAGAAAGTAATATCATGCGTCAATCGTAT<br>GTGAATGCTGGTCGCTATACTG**
**HIS3MX6**	**GACATGGAGGCCCAGAATACCCTCCTTGACAGTCTTGACG<br>TGCGCAGCTCAGGGGCATGATGTGACTGTCGCCCGTACAT<br>TTAGCCCATACATCCCCATGTATAATCATTTGCATCCATACA<br>TTTTGATGGCCGCACGGCGCGAAGCAAAAATTACGGCTCC<br>TCGCTGCAGACCTGCGAGCAGGGAAACGCTCCCCTCACA<br>GACGCGTTGAATTGTCCCCACGCCGCGCCCCTGTAGAGA<br>AATATAAAAGGTTAGGATTTGCCACTGAGGTTCTTCTTTCA<br>TATACTTCCTTTTAAAATCTTGCTAGGATACAGTTCTCACA<br>TCACATCCGAACATAAACAACCATGGGTAGGAGGGCTTTT<br>GTAGAAAGAAATACGAACGAAACGAAAATCAGCGTTGCCA<br>TCGCTTTGGACAAAGCTCCCTTACCTGAAGAGTCGAATTT<br>TATTGATGAACTTATAACTTCCAAGCATGCAAACCAAAAG<br>GGAGAACAAGTAATCCAAGTAGACACGGGAATTGGATTCT<br>TGGATCACATGTATCATGCACTGGCTAAACATGCAGGCTG<br>GAGCTTACGACTTTACTCAAGAGGTGATTTAATCATCGAT<br>GATCATCACACTGCAGAAGATACTGCTATTGCACTTGGTAT<br>TGCATTCAAGCAGGCTATGGGTAACTTTGCCGGCGTTAAA<br>AGATTTGGACATGCTTATTGTCCACTTGACGAAGCTCTTT<br>CTAGAAGCGTAGTTGACTTGTCGGGACGGCCCTATGCTGT<br>TATCGATTTGGGATTAAAGCGTGAAAAGGTTGGGGAATTG<br>TCCTGTGAAATGATCCCTCACTTACTATATTCCTTTTCGGT**

	**AGCAGCTGGAATTACTTTGCATGTTACCTGCTTATATGGTA GTAATGACCATCATCGTGCTGAAAGCGCTTTTAAATCTCT GGCTGTTGCCATGCGCGCGGCTACTAGTCTTACTGGAAGT TCTGAAGTCCCAAGCACGAAGGGAGTGTTGTAAAGGATA CTGACAATAAAAAGATTCTTGTTTTCAAGAACTTGTCATTT GTATAGTTTTTTTATATTGTAGTTGTTCTATTTTAATCAAAT GTTAGCGTGATTTATATTTTTTTTCGCCTCGACATCATCTG CCCAGATGCGAAGTTAAGTGCGCAGAAAGTAATATCATGC GTCAATCGTATGTGAATGCTGGTCGCTATACTG**

Supplementary figure S1

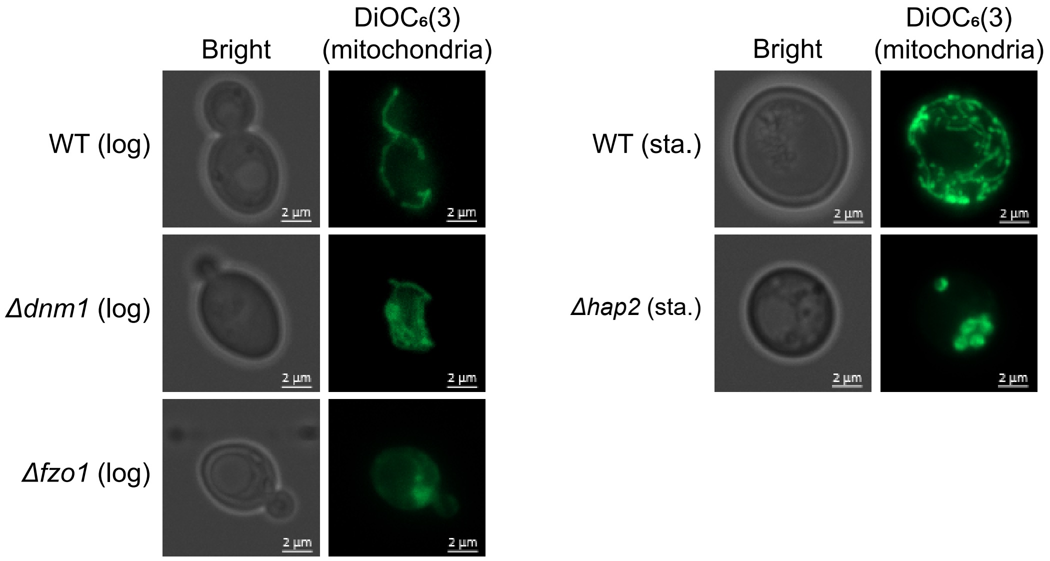

Figure S1. Mitochondria stained with DiOC$_6$(3) viewed under Zeiss Axioskop 2 mot plus fluorescence microscope.
Mitochondria from WT, *Δdnm1*, *Δfzo1*, and *Δhap2* in log or stationary phase were labelled with DiOC$_6$(3) and imaged using a Zeiss Axioskop 2 mot plus fluorescence microscope.

Supplementary figure S2

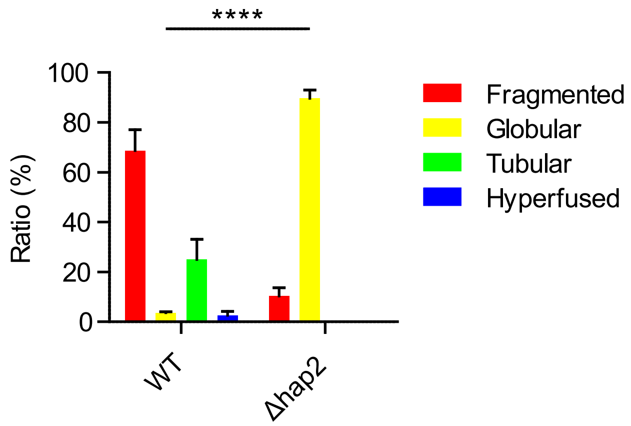

Figure S2. Majority of *Δhap2* cells contain globular mitochondria.
Mitochondria of WT and *Δhap2* in the stationary phase were labeled with preSu9-EG-FP and classified by fluorescence microscopy. Mitochondrial morphology was divided into four categories: fragmented, globular, tubular, and hyperfused. Classification results for WT and *Δhap2* were collecteded from 3 independent experiments (n > 200 cells per experiment). Statistical analysis was based on two-way ANOVA. ****: *p* value < 0.0001.
